# WHAT DO NURSES NEED TO IMPLEMENT FUNCTION-FOCUSED CARE IN THE HOSPITAL?

**DOI:** 10.1093/geroni/igad104.1110

**Published:** 2023-12-21

**Authors:** Marie Boltz

**Affiliations:** Pennsylvania State University, State College, Pennsylvania, United States

## Abstract

The intervention, FFC-AC-EIT, is a theoretically based intervention that includes the implementation of four steps: (1) Environment and policy assessments; (2) Education of staff; (3) Establishing patient goals; and (4) Mentoring and motivating of staff, patients, and families. The nurse interventionist works with a unit-based stakeholder team to develop function-focused goals for the unit and an action plan to meet the goals, while considering barriers to implementation. Based on content analysis of field notes we will describe stakeholder goals established, action plans, and barriers and facilitators to meeting goals in five hospital units. Goals focused on improving staff competency in increasing mobility and physical activity in persons with dementia, integrating mobility assessment and plans into workflows, and improving patient outcomes including decreased falls and less use of constant observation. Common barriers included unstable staffing, inconsistent prioritization among staff and managers, family attitudes, and lack of operating procedures and resources to provide function-focused care. Facilitators at the unit level included administrative support, nurse managers being actively involved in all aspects of planning and oversight, engagement of families, effective communication strategies, involvement of nursing assistants, interdisciplinary collaboration, and access to necessary supplies and equipment. Key implementation strategies included multiple approaches to staff training and communication, the use of champions, clear operating procedures, hardwiring assessment and plans into the electronic record, the use of a feedback loop, and alignment of functional-focused care with other hospital and unit quality initiatives.

